# Editorial: Nutrition and prevention of Alzheimer's disease

**DOI:** 10.3389/fnagi.2015.00170

**Published:** 2015-09-03

**Authors:** Claudia Perez-Cruz, Sofía Díaz-Cintra

**Affiliations:** ^1^Departamento de Farmacología, Centro de Investigaciones y Estudios Avanzados, CINVESTAVCiudad de México, Mexico; ^2^Departamento de Neurobiología de Desarrollo y Neurofisiología, Instituto de Neurobiología, Universidad Nacional Autónoma de MéxicoQueretaro, Mexico

**Keywords:** obesity, cognition, food, bio-active, ketone, brain, risk factors

The era of industrialization has revolutionized our societies, allowing the emergence of technology and improving our standard of living; however, after almost 200 years we are also experiencing unwanted consequences. Mass production allows us to acquire goods more cheaply, but the quality of products is often compromised: industrial food processing, for example, has developed strategies to offer highly caloric and sweeter food by adding low cost-ingredients (i.e., palm oil, high-fructose corn syrup), whose consumption correlates with the development of obesity (O'Kane, 2012). In addition, sedentary life-styles and stressful workloads influence the rates of chronic degenerative diseases such as diabetes, cardiovascular disease, and metabolic pathologies. Over the last decade, many researchers have shown that metabolic disturbances such as obesity and metabolic syndrome are risk factors for the development of dementias and even Alzheimer's disease (AD) (Solfrizzi et al., 2011; Emmerzaal et al., 2015). The mechanisms involved in the AD pathogenesis associated with metabolic alterations are now becoming more understandable. In spite of this and due to the multifactorial complexity of the pathology, more preclinical and clinical data are still needed to understand its development.

An optimal supply of nutrients is necessary to maintain normal functioning of the brain. Mechanistic studies, epidemiologic analyses, and randomized intervention trials have provided insight into the positive effects of micronutrients (vitamins B, E, C, and D) as well as docosahexaenoic acid (DHA) in helping neurons to cope with aging (Wengreen et al., 2013). As a result, considerable attention has been given to nutraceuticals and nutritionally bioactive compounds that can be obtained directly from the diet or supplements and have been reported to have positive effects in animal models of AD and obesity. As an example, Concord grape juice have shown cognitive enhancing effects in subjects with mild-cognitive-impairment (Krikorian et al., 2010a), while blueberry supplementation has improved memory performance in older adult (Krikorian et al., 2010b). However, more randomized controlled clinical trials are needed to determine whether food or food supplements can slow or prevent age-related cognitive decline or AD. The aim of this *research topic* is to cover a range of publications about the impact of poor nutrition (overweight, obesity) on the development of AD and to highlight the use of nutrition as a preventive strategy to reduce the incidence of dementias and AD. Here we describe briefly, the articles included in this research topic.

Hsu and Kanoski have reviewed studies on the effect of the Western diet on the blood-brain-barrier (BBB) and cognitive decline. These authors have shown that the Western diet causes BBB disruption, and they propose this damage as one of the main factors responsible for the cognitive impairments seen in obese animals (Hsu and Kanoski, 2014).

Panza et al. open a discussion about the term “cognitive frailty” and the vulnerability of patients with mild cognitive and other pre-dementia syndromes to develop AD. Macro- and micronutrient deficiencies have been related to frailty in older subjects, whereas nutrition and physical exercise may improve cognition in frail and pre-frail states (Panza et al., 2014).

Swaminathan and Jicha postulated in a comprehensive review that nutrition is a disease-modifying approach against AD development, based on preclinical and clinical studies (Swaminathan and Jicha, 2014). In an opinion article, Santos et al. describe the role of selenium (Se) in the antioxidant system, and they discuss clinical and preclinical data showing that low plasma levels of Se are associated with higher AD incidence (Santos et al., 2014).

Micronutrients such as polyphenols have important anti-oxidant, anti-inflammatory, and anti-carcinogenic activities. Resveratrol is a polyphenol present in grapes, and Wang et al. have tested its anti-amyloid properties in a mouse model of AD. The efficacy of resveratrol and other grape extracts was assessed in a cognitive task, and Abeta load or Abeta-mediated synaptic toxicity was evaluated by LTP induction in J20 transgenic mice. Resveratrol did not show beneficial effects on these parameters, but grape extracts did. Pasinetti's group suggested that a combination of several bioactive metabolites in the juice extract, not resveratrol alone, might ameliorate AD pathology (Wang et al., 2014).

Impaired glucose metabolism in some regions of the brain has been observed in AD patients (Landau et al., 2012), while a supplement of ketone bodies seems to revert this metabolic alteration and improve cognitive performance in animal models of AD (Kashiwaya et al., 2013). In this issue Farah reports a clinical case study of the effect of caprylic triglyceride, a medium-chain triglyceride that induces ketosis, in a 70-year-old man with probable mild AD. The results provided supporting evidence that ketogenic treatments were cognitively effective in AD (Farah, 2014). However, larger randomized clinical trials on patients diagnosed with AD are desirable.

Qian and Ke reviewed the broad mechanism of action of Huperzine (Hurp A) as an anti-AD agent, in addition to its “cholinergic” mechanism of action, including clinical trials where Hurp A treatments resulted in cognitive improvements in patients with mild- and moderate-AD patients (Qian and Ke, 2014).

Hypo-vitaminosis has been related to cognitive decline in the elderly population, and low vitamin D levels are critically associated with AD (Balion et al., 2012). Annweiler and Beauchet discussed the current threshold that defines vitamin D hypo-vitaminosis. They suggest the threshold be standardized to provide better-directed preventive treatment against cognitive deterioration for those at risk due to low vitamin D (Annweiler and Beauchet, 2014).

AD is a multifactorial pathology, where physiological and life-style parameters may impact brain functioning. Therefore, a holistic diagnostic assessment could offer valuable information to predict progression to dementia. Franke and collaborators provide evidence of the recent *BrainAGE* approach to predict brain aging and risk of developing AD. Based on magnetic resonance imaging data, the authors explore the impact of physiological and clinical data on the risk to develop AD in cognitively unimpaired adults. This article provides further evidence that metabolic changes have an impact on cognitive status, and it proposes the *BrainAGE* approach as an early diagnostic tool to assess the risk of developing dementia based on structural and functional brain aging in conjunction with metabolic parameters (Franke et al., 2014).

Thus, accumulated evidence from preclinical and clinical studies about the neuroprotective effects of macro- and micro-nutrients, as well as whole food and bioactive products suggest a path for further treatment strategies to combat this devastating neurodegenerative disease. A holistic diagnostic approach to detect early alterations plus preventive treatment could reduce the incidence of AD in our current industrialized world.

## Conflict of interest statement

The authors declare that the research was conducted in the absence of any commercial or financial relationships that could be construed as a potential conflict of interest.
